# An Adaptive Deconvolution Method with Improve Enhanced Envelope Spectrum and Its Application for Bearing Fault Feature Extraction

**DOI:** 10.3390/s24030951

**Published:** 2024-02-01

**Authors:** Fengxia He, Chuansheng Zheng, Chao Pang, Chengying Zhao, Mingyang Yang, Yunpeng Zhu, Zhong Luo, Haitao Luo, Lei Li, Haotian Jiang

**Affiliations:** 1 School of Mechanical Engineering, Shenyang Jianzhu University, Shenyang 110168, China; abfxhe@163.com (F.H.); zhaochengying0223@163.com (C.Z.); 2 School of Mechanical Engineering and Automation, Northeastern University, Shenyang 110819, Chinazhluo@mail.neu.edu.cn (Z.L.); lileineu@126.com (L.L.); 3 School of Engineering and Material Science, Queen Mary University of London, London E1 4NS, UK; yunpeng.zhu@qmul.ac.uk; 4 Key Laboratory of Robotics, Shenyang Institute of Automation, Chinese Academy of Sciences, Shenyang 110016, China; 5 School of Mechanical Engineering, Dalian University of Technology, Dalian 116024, China

**Keywords:** bearing faults, fault separation, IES-CYCBD, complex faults, fault diagnosis

## Abstract

To address the problem that complex bearing faults are coupled to each other, and the difficulty of diagnosis increases, an improved envelope spectrum–maximum second-order cyclostationary blind deconvolution (IES-CYCBD) method is proposed to realize the separation of vibration signal fault features. The improved envelope spectrum (IES) is obtained by integrating the part of the frequency axis containing resonance bands in the cyclic spectral coherence function. The resonant bands corresponding to different fault types are accurately located, and the IES with more prominent target characteristic frequency components are separated. Then, a simulation is carried out to prove the ability of this method, which can accurately separate and diagnose fault types under high noise and compound fault conditions. Finally, a compound bearing fault experiment with inner and outer ring faults is designed, and the inner and outer ring fault characteristics are successfully separated by the proposed IES-CYCBD method. Therefore, simulation and experiments demonstrate the strong capability of the proposed method for complex fault separation and diagnosis.

## 1. Introduction

Due to bearing failures causing huge property and personnel losses, the analysis of bearing fault features has always been an important problem in the engineering field. With the improvement in researchers’ understanding of the time domain, frequency domain and statistical parameters of vibration signals, multiple mathematical tools and signal processing methods have been developed to address the problem of fault feature extraction and diagnosis. However, the components of the actual vibration signal are complex, and the existing signal processing methods have a poor performance when dealing with high noise and frequency interference of vibration signals. The improvement and innovation of bearing fault feature extraction and detection methods is the focus of this paper.

When a bearing fails, the contact force between the rolling element and the raceway undergoes a sudden change. Under the influence of the load on the bearing, time-domain fault features dominated by impact excitation will be generated in the vibration acceleration signal. At this point, the vibration response level of the bearing significantly increases compared to normal operating bearings. Meanwhile, the vibration amplitude and frequency domain features of the acceleration signal are affected by the rotor motion state. Therefore, dynamic modeling research into faulty bearings needs to be combined with rotor modeling. Liew et al. [1] established a five-degrees-of-freedom bearing dynamics model, where the inner ring contains three degrees of freedom for radial and axial displacement, as well as two degrees of freedom for rotational displacement. Qiu et al. [2] established a stiffness-based prognostics model for a bearing system based on vibration response analysis and damage mechanics to predict the remaining life of an operating bearing. Ahmadi et al. [3] modeled the finite size and mass of rolling elements to more accurately predict the time taken for rolling elements to enter and exit the defects. Zmarzły [4] proposed the concept of technological heredity and operational (exploitation) heredity in the production and operation of rolling bearings. Based on an experimental study, an operation heredity analysis of ball bearings has been carried out. Huang et al. [5] established a coupled dynamic model of a flexible rotor bearing system and investigated the resonance effect of a rotor system supported by rolling bearings due to the nonlinearity of the bearings, which is more pronounced when the bearings have defects.

Maximum second-order cyclostationary blind deconvolution (CYCBD) is a blind deconvolution method based on the generalized Rayleigh quotient. The principle of this method is to maximize the second-order stationarity of vibration signals by adjusting the deconvolution filter. Even if the pulse noise or velocity is not constant, the recovery ability of the pulse cyclic stationary source is stronger than that of other deconvolution methods. Cyclostationarity can be used to discover hidden cyclostationarity features caused by faults in signals [6]. Marco [7] first proposed the CYCBD method and applied this method to the diagnosis of gear tooth crack faults and bearing outer ring faults. Wang [8] combined the cuckoo search algorithm (CSA) with CYCBD to optimize the loop frequency and filter length parameters. Chen et al. [9] proposed periodic detection techniques (PDTs) to identify the cycle of repetitive pulses in response to the limitations of the deconvolution algorithm. The improved deconvolution method can automatically identify fault cycles using PDTs based on the features of the measured signal, and can also adaptively enhance pulse signals from different faults.

At present, when extracting bearing fault features, the Hilbert transform is generally used to demodulate the signal first, and then analyze the envelope spectrum of the demodulated signal. The square envelope spectrum (SES) is mainly composed of the spectrum of the transformed square envelope (SE) of the signal. To enhance its detection capabilities, the signal can be filtered around the excitation frequency band containing fault information. The filtered signal has a high signal-to-noise ratio (SNR) and more obvious fault features [10]. Therefore, the focus of research on frequency band localization methods has become bearing fault diagnosis. At present, the most effective optimal frequency band localization method is the Fast Kurtogram, which is an automatic frequency band selection tool based on maximum kurtosis [11]. Through bandpass filtering, the vibration signal is divided into one-third binary trees according to different center frequencies and bandwidths, searching for the optimal resonance frequency band interval. In addition, Moshrefzadeh [12] developed a different frequency band selection tool to obtain the SES. An important conclusion from this method was that the frequency band selection tool only chose one node as the optimal node, while other unused nodes might typically contain ignored valid information. Tse et al. [13] mainly analyzed the sparsity of ultrasonic signals and utilized the sparsity levels of different frequency bands based on wavelet packets to detect high resonance frequency bands and amplify the fault signal of bearings. Antoni et al. [14] utilized the negative entropy of signals for frequency band localization and applied the definition of entropy in thermodynamics to reflect the degree of signal deviation from equilibrium state. Smith et al. [15] proposed a method based on spectral kurtosis to select the optimal demodulation frequency band and extract fault related pulse components from vibration signals contaminated by strong electromagnetic interference. Some scholars believed that extracting kurtosis from the envelope signal rather than the original signal will yield a better positioning effect [16].

After frequency band localization, envelope spectrum needs to be used to demodulate and analyze the bandpass-filtered signal. In recent years, cyclic spectral correlation (CSC) and cyclic spectral coherence (CSCoh) have been widely used as fault signal demodulation and analysis tools that can replace SES in bearing fault detection [17,18,19]. The advantage of these methods is that they can explain the hidden cycle of second-order cyclostationarity in the signal under stronger interference. By integrating the spectral axis of the bivariate mapping in the frequency domain, an enhanced envelope spectrum (EES) or an improved envelope spectrum (IES) can be obtained. Kilundu [20] described the cyclostationarity features of acoustic emission signals from defective bearings, and pointed out that indicators based on cyclostationarity technology are more sensitive to continuous defect detection compared to traditional time indicators (RMS, kurtosis, and peak factor). Although the IES method has outstanding capabilities in the demodulation of fault pulses, the selection of the optimal integration band affects the level of fault feature frequency relative to background noise in the envelope spectrum. To address this limitation, Mauricio [21] proposed a method of improving envelope spectrum through feature optimization graphs (IESFOgram) as a frequency band selection tool for bivariate mapping (CSC or CSCoh), and demonstrated that this method outperforms fast kurtogram and autogram in terms of performance. In subsequent research on the IESFogram method, the envelope spectrum fault eigenvalues which correspond to the maximum cyclic frequency were used to describe the spectral density of a specific fault frequency, and the optimal frequency band was selected based on the maximum value of this function. Multiple frequency bands were combined to allow the most amount fault feature information to be contained within the envelope spectrum [22,23]. Xiao [24] analyzed the original vibration signal through a fast spectrum and used the kurtosis-enhanced spectral entropy (KESE) index to locate the fault frequency band from the entire frequency band, thereby highlighting the fault excitation pulse.

The above diagnostic methods focus on rolling bearings with single point faults. In practice, when defects occur, bearing failures often manifest as a compound fault due to the existence of debris and impurities. Due to the complex operating environment, the interaction of multiple noise sources, and the phenomenon of mutual coupling and interference between composite faults, the difficulty of detecting composite faults is greater than that of single fault diagnosis. Moreover, decoupling composite fault features under strong noise background is a challenge in the field of fault diagnosis [25]. Jiang et al. [26] proposed a decoupling diagnosis method for rolling bearing composite faults based on the empirical wavelet transform duffing oscillator (EWTDO). The method uses empirical wavelet transform to extract the inherent modes of the signal and decompose the composite faults into different single faults in the form of empirical modes. Hao et al. [27] proposed a sparse component analysis method based on three-dimensional geometric features (TGF-SCA), which successfully separates and extracts bearing faults without pre-determining the number and frequency of the original signals. Xu et al. [28] used a combination of fast empirical wavelet transform and spectral entropy to construct feature optimization information maps, selecting the optimal frequency band center and bandwidth for bandpass filtering. Lyu et al. [29] used the quantum genetic algorithm (QGA) to optimize the parameters of the MCKD algorithm, perform power spectrum analysis on gear signals, and analyze the envelope spectrum of bearing fault signals. Tang et al. [30] proposed a composite fault separation method based on singular negentropy difference spectrum (SNDS) and integrated fast spectral correlation (IFSC), which first denoised the fault signal and then separated the fault features. Meng et al. [31] used periodic weighted kurtosis (PWK) to solve the problem of kurtosis only measuring transient characteristics when evaluating repetitive pulses. Lotfi [32] proposed a new bearing diagnosis pattern classification method that combines high-order spectral analysis features and support vector machine classifiers. This method performs principal component analysis to reduce the dimensionality of the extracted bispectral features. Manjurul [33] proposed a multi combination fault diagnosis scheme based on an improved multi classification support vector machine (MCSVM) and extracted bearing fault features using acoustic emission signals. Dhiman [34] used neighborhood component analysis (NCA) to select the best features, and the results showed that NCA can effectively improve the accuracy and reliability of monitoring systems. Zhou [35] proposed a feature extraction method based on NCA to reduce the dimensionality of the original feature set, so as to avoid the redundancy caused by excessive dimensionality and the decrease in diagnostic accuracy. As mentioned above, the research on feature extraction methods for composite faults in bearings has gradually become a research hotspot because it is more in line with the actual working conditions of bearings. Poor working conditions lead to the occurrence of bearing faults that are not singular. Multiple fault components interfere with each other, frequency components are complex, noise intensity is high, and other reasons cause severe difficulties in fault separation, making it judging the working condition of bearings inaccurate.

Due to the coupling of vibrations from different faults, the fault features of the vibration signal become more complex, increasing the difficulty of bearing fault diagnosis. So far, various signal processing methods have been used for composite fault diagnosis in bearings, including empirical mode decomposition [36], variational empirical mode decomposition [37], wavelet transform [38], etc. These signal processing methods decompose the signal into different frequency bands according to adaptive methods. Although they achieve the diagnosis of composite faults in bearings, the lack of purposefulness in frequency band segmentation during the signal decomposition process results in unsatisfactory segmentation results. Therefore, these decomposition methods limit our ability to diagnose composite faults. In summary, it is necessary to study the decoupling method for extracting bearing composite fault features that can more accurately segment frequency bands and perform demodulation analysis on them. Therefore, this paper proposes a bearing fault diagnosis method with a higher classification accuracy based on the cyclostationarity of bearing fault signals.

Moreover, the basic knowledge about CYCBD and IES is reviewed in Section 2. In Section 3, the proposed method, named IES-CYCBD and based on feature optimization, is illustrated in detail. Section 4 and Section 5 demonstrate the ability of the proposed method by simulation and experimental cases. Finally, conclusions are drawn in Section 6.

## 2. Theoretical Background

The blind deconvolution method mainly recovers periodic fault shocks ***s*** from the noise signal ***x*** generated by the convolution of the transmission path ***g***; this method can be expressed as

(1) $$s=x*h=\left(s_{0}*g\right)*h\approx s_{0}$$

(2) $$x=s_{0}*g_{s}+p*g_{p}+n*g_{n}$$

where ***g_s_***, ***g_p_***, and ***g_n_*** represent impulse response functions related to ***s***_0_, ***s***_0_ represents periodic impacts caused by local faults, ***p*** represents periodic components such as turnover frequency that are not related to faults, and ***n*** represents Gaussian noise caused by transmission paths and sensors. Hence, the deconvolution method can be described as separating the impulse excitation components caused by local faults from the test signal through a FIR filter; the deconvolution method can be expressed as

(3) $$s=\left(s_{0}*g_{s}+p*g_{p}+n*g_{n}\right)*h\approx s_{0}$$

It should be noted that many deconvolution methods cannot reflect the amplitude of the original signal, only the waveform features of the signal to be separated. This feature satisfies a certain statistical information source of the signal and can be used to extract bearing fault features. When calculating the optimal filter ***h***, using a reasonable optimization objective function is the key to blind deconvolution method for separating fault signals. Kurtosis can better reflect the size of the impact components in the signal, so the kurtosis index can be used as the objective function in the blind deconvolution method (MED). But, when subjected to external random shocks, this indicator will be misjudged as a fault shock signal. In order to address the limitations of this method, various indicators have been proposed, such as correlation kurtosis (MCKD), multiple norm (MOMED), pulse norm, average kurtosis, autocorrelation pulse harmonic noise, etc. However, these indicators have not shown the statistical characteristics of the cyclostationary process of the signal itself.

### 2.1. Maximum Second-Order Cyclostationarity Blind Deconvolution

The vibration signal of rotating machinery can be regarded as a mixed signal of first-order and second-order cyclostationarity processes, where the cyclic frequency can be regarded as a frequency related to a certain fluctuation of signal energy, and the second-order cyclostationarity index in the discrete form of the signal can be expressed as

(4) $${\mathrm{ICS}}_{2}=\frac{|s{|}^{2H}EE^{H}|s{|}^{2}}{{\left|s^{H}s\right|}^{2}}$$

(5) $$\left\{ \begin{array}{l} |s{|}^{2}={\left[|s[N-1]{|}^{2},\ldots ,|s[L-1]{|}^{2}\right]}^{T}\\ E=\left[\begin{array}{lllll} e_{1} & \cdots & e_{k} & \cdots & e_{K} \end{array}\right]\\ e_{k}={\left[e^{-j2\pi \alpha_{k}(N-1)},\ldots ,e^{-j2\pi \alpha_{k}(L-1)}\right]}^{T} \end{array}\right.$$

where *α_k_* represents the cyclic feature frequency, and the cyclic feature frequency group is defined as

(6) $$\alpha_{k}=\frac{K}{T_{s}},\left(K=1,2,\ldots ,N\right)$$

where *T_s_* represents the interval time between impact signals caused by defects. The process of maximizing the statistical components of the constituents related to the characteristic cyclic frequency in the signal is to solve the optimal filter bank ***h****_o_* by taking the characteristic frequency of bearing faults as the cyclic frequency of the discrete time signal. Therefore, the second-order stability index can be transformed into

(7) $${\mathrm{ICS}}_{2}=\frac{h^{H}X^{H}WXh}{h^{H}X^{H}Xh}=\frac{h^{H}R_{XWX}h}{h^{H}R_{XX}h}$$

(8) $$W=\left[\begin{array}{lll} \ddots & & 0\\ & \mathbb{P}\left[|s{|}^{2}\right] & \\ 0 & & \ddots \end{array}\right]\frac{\left(L-N+1\right)}{\sum_{l=N-1}^{L-1}{\left|s\right|}^{2}}$$

(9) $$\mathbb{P}[s]=\frac{EE^{H}|s{|}^{2}}{L-N+1}$$

where ***R_XWX_*** and ***R_XX_*** represent the weighted correlation matrix and correlation matrix, respectively, transforming the maximization problem of ICS_2_ on ***h*** into the eigenvector corresponding to solving the maximum eigenvalue.

(10) $$R_{XWX}h=R_{XX}h\lambda$$

The maximum λ corresponding to the maximum value of ICS2 is obtained by the following iterative Algorithm 1:
**Algorithm 1.** CYCBD algorithm.Input: test signal *x*, filter length *L*, bearing fault characteristic frequency *f_fault_* Initialize filter bank ***f*** = [0, 0, ⋯, 1, −1, ⋯, 0, 0]*^T^*, initial error ***ε*** = 0, maximum iterations 30While: relative error less than threshold or maximum iterations reached, ***ε*** < ***ε***_0_ or *iter* < 30 Step1: calculate the cyclic characteristic frequency group *α* weighted matrix *W* Step2: calculate matrices ***R_XWX_*** and ***R_XX_*** to solve eigenvalue problems Step3: the deconvolution filter ***f*** is updated with ***R_XWX_h*** = ***R_XX_h****λ* Step4: calculate the relative error of the maximum eigenvalue of adjacent iterations ***ε***. EndOutput: optimal deconvolution signal *y*

CYCBD demonstrates a more accurate performance than other deconvolution methods in diagnosing impact signals of early bearing faults. However, when using the CYCBD method to recover bearing fault impact signals, accurate parameters need to be set. Among them, increasing the filter length parameter will enhance the filtering effect, but it requires more computational resources. Differently to the MCKD method, adjusting to a higher numerical size can ensure that ideal results can be obtained. Compared to the filter length parameter, the fault related cycle frequency is more important. In the actual process of bearing fault diagnosis, due to the sliding factor of the rolling element of the bearing and the fluctuation in the shaft rotation frequency, the frequency calculated by the physical size of the bearing may deviate from the actual bearing fault characteristic frequency, which limits the application of the CYCBD method in separating periodic impact signals.

### 2.2. Improved Envelope Spectrum

A cyclostationary process is a periodic behavior process that displays its statistical characteristics, which can accurately reflect the vibration signal characteristics of rotary machines [23]. Real mechanical signals are usually composed of first-order and second-order cyclic processes. During the signal acquisition process, rotating mechanical components may generate periodic cyclic transient signals, which typically carry information related to the health status of mechanical components. Therefore, it is possible to detect bearing faults and track the evolution of defects by analyzing the cyclostationarity of the signal. To obtain signals of interest from rotating machinery, the first two orders of cyclostationary signals can be used to characterize them, the first order cyclostationary signal *C*_1*x*_(*t*) can be expressed as

(11) $$C_{1x}\left(t\right)=E\left[x\left(t\right)\right]=C_{1x}\left(t+T\right)$$

where E[·], *t*, and *T* stand for the set averaging operator, time, and period, respectively. The first order cyclostationary (CS1) characteristics of the signal mainly come from component vibrations related to rotor rotation frequency (such as shaft misalignment, meshing gear peeling, etc.). The second-order cyclostationary (CS2) feature represents the periodicity of the second-order statistical moments of the signal, which can be expressed as

(12) $$C_{2x}\left(t,\tau \right)=E\left[x\left(t\right)x{\left(t-\tau \right)}^{*}\right]=C_{2x}\left(t+T,\tau \right)$$

where *τ* and * represent as a delay variable and conjugate operation, respectively.

Cyclic spectral correlation (CSC) is a tool that can describe CS1 and CS2 information. This method is represented as a distribution function of two frequency variables: cyclic frequency *α* related to modulation and the spectral frequency *f* associated with the carrier signal. This tool can also describe the correlation distribution between the carrier and modulation frequencies present in the frequency components of the signal, and it is defined as

(13) $$\mathrm{CSC}\left(\alpha ,f\right)=\underset{W\to \infty }{\lim}\frac{1}{W}E\left[F_{W}\left[x\left(t\right)\right]F_{W}{\left[x\left(t+\tau \right)\right]}^{*}\right]$$

where F*_W_*[*x*(*t*)] represents the fast Fourier Transform of signal *x*(*t*) in finite time *W*. Processing CSC can obtain a bivariate mapping that reveals hidden modulation frequencies. In order to reduce uneven distribution, the CSC was normalized to obtain cyclic spectral coherence (CSCoh), which is used to describe the normalized values of spectral correlation between 0 and 1. The cyclic spectral coherence is calculated by the following formula

(14) $$\mathrm{CSC}\mathrm{oh}(\alpha ,f)=\frac{\mathrm{CSC}(\alpha ,f)}{\sqrt{\mathrm{CSC}(0,f)\mathrm{CSC}(0,f+\alpha )}}$$

Integrating along the spectral frequency axis can obtain a one-dimensional spectral function of the cyclic frequency. In the cyclic spectrum of spectrum frequency integration, the frequency band can be defined as the complete available frequency band from 0 to the Nyquist frequency, thereby displaying the components of all the modulation frequencies present in the signal. Therefore, enhanced envelope spectrum (EES) can be used as a tool for demodulating fault signals. It is defined as

(15) EES(α)=∫|CSCoh(α,f)|df

The fault frequency characteristics related to the current damage in the signal can be enhanced by integrating a specific frequency band on a bivariate map. The resulting spectrum is called Improved Envelope Spectrum (IES); it can be calculated by the following formula

(16) $$\mathrm{IES}(\alpha )=\int_{f_{2}}^{f_{1}}\left|\mathrm{CSCoh}(\alpha ,f)\right|df$$

where *f*_1_ and *f*_2_ represent the upper and lower limits of the integrated frequency band, respectively.

## 3. Proposed Method (IES-CYCBD)

The frequency band selection method used in this paper detects cyclic modulation carriers of interest in bivariate mapping. This method is realized by improving the envelope spectrum of feature optimization, and mainly optimizes the significance of fault feature frequency according to the cyclic features (bearing fault feature frequency) of fault signals on the demodulation spectrum generated by integral bivariable mapping. The resolution of the cycle frequency *α* is the reciprocal of the signal length, and the resolution of the spectral frequency is 1/2 of the sampling frequency *f_s_* divided by the window size defined in the bivariate mapping. The main process of the FOgram method is as follows:

Step1: Extracting bivariate maps from signals. The fast cyclic spectrum method (FastCS) can be used to quickly and accurately obtain bivariate mapping images, generate cyclic spectral correlation (CSC) images, and normalize them to obtain cyclic spectral coherence images (CSCoh) [18]. It is a bivariate image of the loop variables *α* and the spectral variable *f*. In addition, we must define the fault feature ratio (FFR) to reflect the significance of the fault feature frequency in the filtering results, which can be expressed as follows

(17) $$\;FFR=\frac{\frac{1}{I}\sum_{i=1}^{I}A_{f}}{E\left[AS_{t}\right]}$$

(18) $$A_{f}=\max\left(if_{m}-0.02f_{m},if_{m}+0.02f_{m}\right)$$

where *I* is the number of harmonics to be calculated and *AS_t_* is the sum of envelope spectrum amplitudes within the selected frequency range. E[*x*] is used to calculate the mean of signal *x*. *A_f_* is the amplitude corresponding to the envelope spectrum at the fault characteristic frequency. This indicator mainly reflects the significance level of fault feature frequency in periodic detection technology under different background noise conditions.

Step2: According to the algorithm of 1/3 binary tree, frequency bands are divided along the frequency axis, which is similar to the fast kurtosis graph. Each branch on a binary tree is determined by a series of bandwidth *b_w_* and center frequency *f_c_*. The lower and upper limits of the *f* frequency band are *f_c_* − *b_w_*/2 and *f_c_* + *b_w_*/2, respectively. Then, each frequency axis *f* in the band is then integrated to obtain a demodulation spectrum IES*_cf,bw_*(*α*).

(19) $${\mathrm{IES}}_{cf,bw}(\alpha )=\int_{cf+bw/2}^{cf+bw/2}|\mathrm{CSCoh}(\alpha ,f)|df$$

Step3: Harmonic product spectra are generated from each demodulation spectrum IES*_cf,bw_*(*α*), and the kurtosis index is extracted. The fault information contained in the resonant frequency band can be found in the enhanced envelope spectrum, and its significance relative to the background noise can be reflected by the spectral kurtosis of the enhanced envelope spectrum *Ku*. This method is different from the diagnostic features calculated from the fault feature frequency proposed by Mauricio [23]. Using the kurtosis index of harmonic product spectrum to calculate the bearing fault feature frequency does not require accurate pre-determination, but only provides a wider frequency detection range. Even if the pre-determined fault feature frequency changes in a small range, the optimal frequency band range can be accurately located. This indicator solves the problem of changing the frequency of bearing faults due to random sliding during actual bearing operation, which leads to the FFR indicator being unable to accurately reflect the severity of faults. The improved envelope spectral kurtosis calculation method within the frequency search range can be expressed as

(20) $$Ku_{cf,bw}=\frac{\frac{1}{N}\sum_{i=1}^{N}{\left({\mathrm{HSI}}_{cf,bw}\left(\alpha_{i}\right)-mean\left({\mathrm{HSI}}_{cf,bw}\left(\alpha_{i}\right)\right)\right)}^{4}}{{\left(\frac{1}{N}\sum_{i=1}^{N}{\left({\mathrm{HSI}}_{cf,bw}\left(\alpha_{i}\right)-mean\left({\mathrm{HSI}}_{cf,bw}\left(\alpha_{i}\right)\right)\right)}^{2}\right)}^{2}}$$

where HSI*_cf,bw_*(*α*) represents the harmonic product spectrum of IES within the selected fault characteristic frequency range in the (*cf*, *bw*) frequency band. However, frequency bands with more discrete frequencies can also increase the kurtosis value of the envelope spectrum, so entropy is used to help evaluate the level of discrete frequency components in the harmonic product spectrum [36]. Therefore, this paper proposes the Optimal Fault Characteristics (OFC) to describe the significance of fault features, it can be defined as

(21) $${\mathrm{OFC}}_{fc,bw}=\frac{Ku_{cf,bw}}{-\sum_{i=1}^{N}E_{i}\log\left(E_{i}\right)}$$

where

(22) $$E_{i}=\frac{{\mathrm{HSI}}_{cf,bw}\left(\alpha_{i}\right)}{\sum_{i=1}^{N}{\mathrm{HSI}}_{cf,bw}\left(\alpha_{i}\right)}$$

Step4: Finding the optimal frequency band for bivariate integration. The existence of cyclic components in each frequency band is quantified through the OFC database formed by calculating OFC values of different center frequencies and frequency band widths in setp3. By now, the optimal frequency band is equivalent to the frequency band corresponding to maximizing the OFC value. In a 1/3 binary tree, the color map corresponding to the OFC size as a function of (*cf*, *bw*) is represented as FOgram, with its maximum value corresponding to the optimal integration frequency band.

(23) $$OB=\underset{cf,bw}{\mathrm{argmax}}[OFC\left(cf,bw\right)]$$

Step5: Finally, the optimal frequency band OB selected by FOgram is integrated along the frequency axis *f* to extract bearing fault features. 

The OFC index is used to select the bearing resonance frequency band caused by bearing fault frequency, which can better remove the interference of irrelevant frequency components and noise on fault characteristic frequency. Compared to other fault impact cycle detection methods such as envelope spectrum autocorrelation [39], periodic modulation intensity (PMI) [40], and other quantitative indicators, the extraction of bearing fault features based on feature optimization envelope spectrum can more accurately avoid noise interference, providing more accurate results for the selection of cyclic feature frequency vectors. 

**Figure 1:**
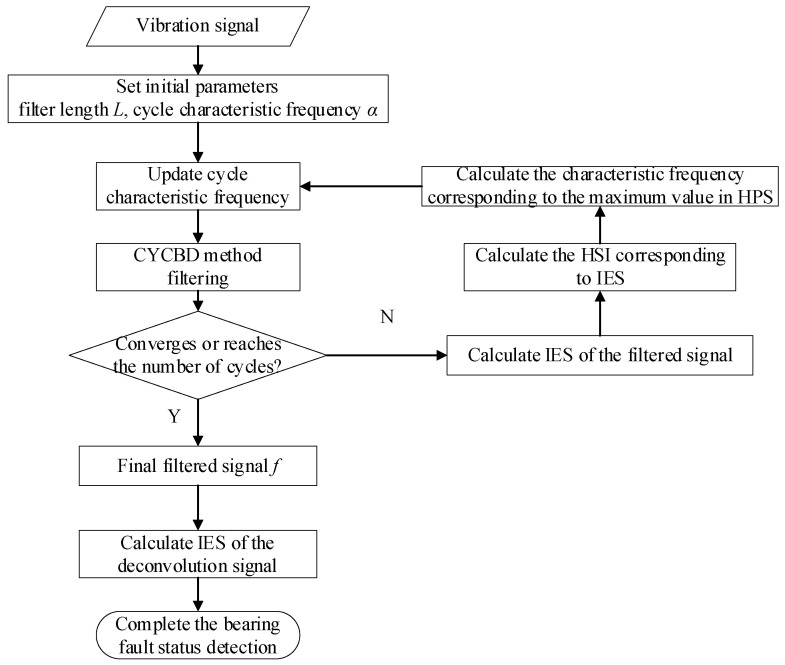
IES-CYCBD algorithm flow.

The specific process of this method is shown in Figure 1. It mainly includes three parts: fault feature frequency search, CYCBD method filtering, and composite fault feature separation.

Firstly, the enhanced envelope spectrum extracted from the resonance frequency band is used to identify the cyclic feature frequency using the harmonic product spectrum, and the recognition result is used as the cyclic feature frequency group of the CYCBD method for deconvolution calculation. Then, when the deconvolution effect is not obvious, the accuracy of identifying fault feature frequencies in low signal-to-noise ratio backgrounds is improved, solving the problem of reduced deconvolution effect caused by the deviation between the preset bearing fault feature frequency and actual working conditions, and enhancing the filtering effect of the CYCBD method. The CYCBD method enhances the frequency component of the resonance frequency band in the signal. Next, feature optimization maps can be used to more accurately locate the resonance frequency band excited by faults in the signal. Finally, the optimized IES is obtained by integrating the frequency axis within the determined frequency band, and different types of bearing fault features included in the signal are separated.

## 4. Numerical Simulation

In this section, the effectiveness of the IES-CYCBD method is verified by simulating bearing fault signals. The simulated fault signal model is shown in Section 4.1, which contains four common signal components. Then, the proposed method (IES-CYCBD) is compared with other methods, and the superiority of the IES-CYCBD method is verified.

### 4.1. Vibration Model for Rolling Element Bearing

The measurement signal *x* consists of the following components: pulses caused by local faults, pulses caused by external random shocks, pure periodic components, and Gaussian background noise. Consider the following assumptions when simulating fault signals: (a) The bearing fault signal *S_f_* caused by local defects independently satisfies identical distribution and has unique statistical characteristics (such as periodic impulsivity). (b) The stationary Gaussian noise *n* and the periodic component *S_p_* are additive, and they do not have the characteristics of the *S_f_* signal.

(24) $$x\left(t\right)=S_{f}\left(t\right)+S_{p}\left(t\right)+S_{r}\left(t\right)+n\left(t\right)$$

where *S_f_* (*t*) is a repetitive pulse caused by a local defect in the bearing, as shown in Equation (25), it can be modeled as an exponential decay mode; in Equation (26) *S_r_* (*t*) is used to simulate random pulses generated by external shocks; *S_p_* (*t*) in Equation (27) represents the interference component generated by shaft rotation, etc.; *n* (*t*) is Gaussian white noise used to simulate the noise impact caused by the transmission path.

(25) $$S_{f}(t)=\sum_{i=1}^{N_{1}}A_{f}(t)e^{-\xi_{f}\left(t-iT_{f}-\delta_{i}\right)}\times \cos\left[2\pi f_{f}\left(t-iT_{f}-\delta_{i}\right)\right]$$

where *N*_1_ and *A_f_* (*t*) are used to simulate the number and amplitude of fault pulses per second, respectively, *T_a_* and *f_f_* represent the time interval between adjacent faults and resonance frequency, respectively, and a damping ratio *ξ_f_* = 1200, *δ_i_* represents the random time delay between two impacts caused by rolling bearing sliding, following the average distribution U(0.01*T_a_*, 0.02*T_a_*). The modeling parameters for inner and outer ring faults are shown in Table 1.

(26) $$S_{r}(t)=\sum_{s=1}^{N_{2}}A_{r}e^{-\xi_{r}\left(t-T_{s}\right)}\times \cos\left[2\pi f_{r}\left(t-T_{r}\right)\right]$$

where the resonance frequency *f_r_* = 1700 Hz, the damping coefficient *ξ_r_* = 800, the number of random pulses *N*_2_ = 3, and *A*r represents the amplitude of the frequency conversion and follows a normal distribution with a standard deviation of 2.5 and a mean of 1. *T_r_* represents the time of random pulse occurrence and follows the average distribution within the signal sampling time.

(27) $$S_{p}(t)=\sum_{p=1}^{N_{3}}A_{p}\sin\left(2\pi f_{p}t+\theta_{p}\right)$$

where the number of periodic harmonics *N*_3_ = 2, which is used to simulate the impact of shaft rotation and disc eccentricity mass in the system. *A* represents the amplitude of the harmonic component and *A*_1_ = *A*_2_ = 0.25, *f*_1_ = 10, *f*_2_ = 20 *f* represents harmonic frequency and *f*_1_ = 10, *f*_2_ = 20, *θ* represents the harmonic phase, and *θ*_1_ = *π*/6, *θ*_2_ = −*π*/3.

**Figure 2:**
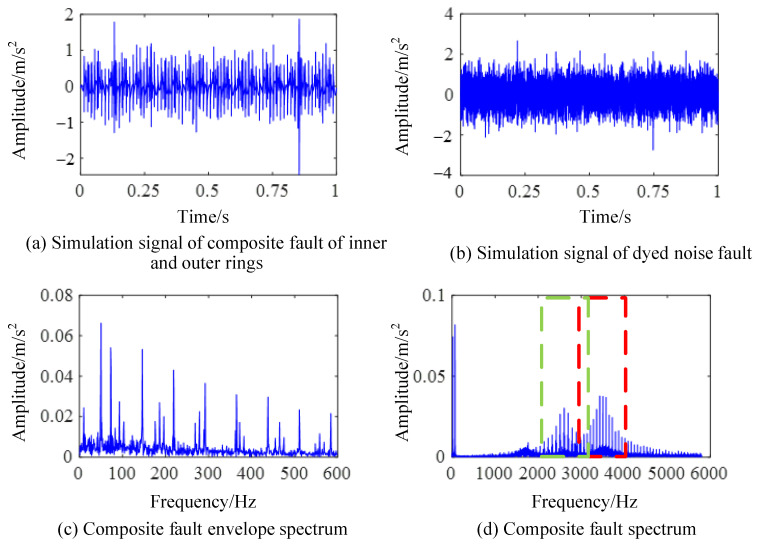
Frequency domain diagram of composite fault simulation signal.

The simulation signal is the sampled signal within 1 s, and the sampling frequency is *f_s_* = 11,904 Hz. The composite bearing fault simulation signal obtained by using the simulated signal as a deconvolution signal is shown in Figure 2. The time-domain simulation of the composite fault signal of the bearing is shown in Figure 2a, which includes an inner ring fault, an outer ring fault, a random impact, and frequency components caused by shaft rotation. In order to verify the fault extraction ability of the fault diagnosis algorithm, a Gaussian noise signal with a standard deviation of 0.6 was added to the bearing fault signal. It can be observed that the attenuated impact signal generated by the fault is masked by noise and difficult to distinguish, as shown in Figure 2b.

The envelope spectrum of the simulated signal is shown in Figure 2c, where the feature frequency of the outer ring fault is 73 Hz and the feature frequency of the inner ring fault is 93 Hz. The corresponding fault feature frequency and its harmonic frequency, as well as the sideband component generated by modulation with a rotation frequency of 10 Hz as the width, can be observed from Figure 2c, which conforms to the inner ring fault feature. Figure 2d shows the spectrum of the signal. The resonance frequency center of the inner ring and the outer ring are set at 2600 Hz and 3500 Hz for the simulation signal. It can be seen from Figure 2d that resonance phenomena in different frequency bands are stimulated by different types of bearing fault shocks in the high frequency region. 

**Figure 3:**
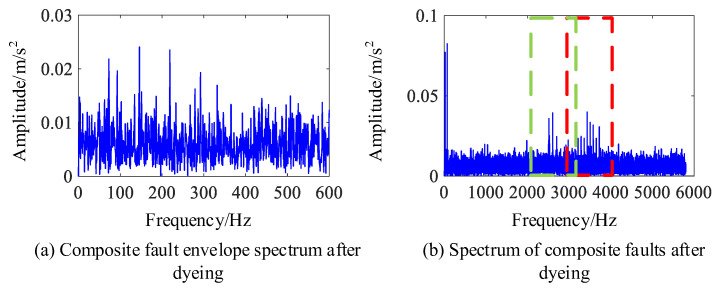
Frequency domain diagram of simulated signal after noise.

The envelope spectrum of the composite fault signal after noise exposure is shown in Figure 3. In this case, only the fault feature frequency of the outer ring can be detected from the envelope spectrum in Figure 3a, while the fault feature frequency of the inner ring cannot be detected when it is masked by the noise frequency. In the spectrum image of the noise-stained signal seen in Figure 3b, the fault information from the bearing is also submerged by the noise in the high-frequency resonance band, which increases the difficulty of fault feature extraction.

### 4.2. Comparison

At present, there are many methods to detect the periodic impacts of bearing faults. Although CYCBD can improve the signal-to-noise ratio of bearing fault signals during the iteration process, searching for fault signal cycles with fewer iterations and more accurate judgment of cycle size can affect the diagnostic effectiveness of CYCBD. Therefore, various cycle estimation methods are analyzed to study the accuracy of fault impact cycle detection under strong background noise using different methods. 

**Figure 4:**
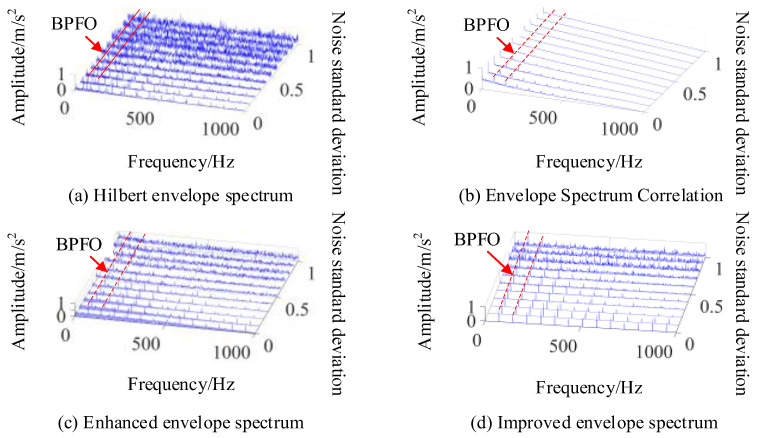
Outer ring fault cycle detection method.

The periodic fault detection methods include the Hilbert envelope spectrum, autocorrelation judgment based on envelope spectrum, the enhanced envelope spectrum method, and the improved envelope spectrum method. In order to compare the effect of frequency estimation under different intensities of noise signal interference, a bearing fault signal with 0–1 standard deviation of Gaussian white noise is added to the fault impact signal. Then, the amplitude of the envelope spectrum is normalized, and the results of the fault cycle detection are shown in Figure 4.

As shown in Figure 4a, the envelope spectrum can accurately reflect an outer circle fault characteristic frequency (BPFO) of 73 Hz and an inner circle fault characteristic frequency (BPFI) of 93 Hz under high signal-to-noise ratio conditions. However, when the noise has a standard deviation which is approximately greater than 0.4, it is difficult to separate the characteristic frequency of the inner ring fault from the noise. When the noise has a standard deviation which is approximately greater than 0.6, the characteristic frequency of the outer ring fault is also submerged in the noise. By using correlation analysis on the envelope spectrum, corresponding correlation coefficients can be obtained. Although the noise signal has a good suppression effect, when using this method it is also difficult to detect the fault feature frequency under the noise cover when the noise intensity increases, as shown in Figure 4b. Figure 4c shows the enhanced envelope spectrum obtained based on CSCoh, which can be used to find more obvious outer circle fault feature frequencies. Under strong noise conditions, the feature frequencies of inner ring faults cannot be separated and the feature frequencies of outer ring faults also become less obvious. Figure 4d shows the enhanced envelope spectrum after feature optimization used in this paper. By setting the range of optimized features to 65~75 Hz, it can be seen that this method separates composite fault features, including the outer circle fault feature frequency within this frequency range, but excluding the inner circle fault feature frequency, which enhances the search performance of the algorithm. Meanwhile, it can be seen from Figure 4d that this method eliminates other frequency interferences unrelated to the target feature frequency under weak noise signals, and has a good suppression effect on noise under strong noise conditions, improving the detection performance of fault cycles. Moreover, it can accurately estimate the frequency of target impacts in the signal without calculating the fault feature frequency. 

**Figure 5:**
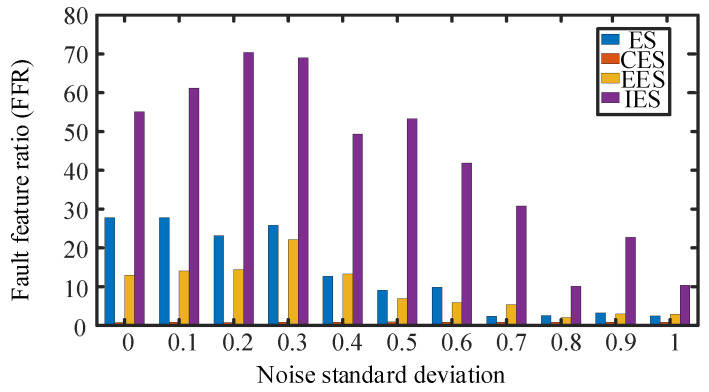
The effect of four periodic detection methods for outer ring faults.

In order to analyze the detection performance of the above methods, the proposed fault feature ratio (FFR) in Section 3 is used as the fault cycle detection indicator. Figure 5 shows that the detection indicators in the four methods decrease when the intensity of the noise signal increases, but IES always maintains a high FFR indicator. The performance of this method is significantly improved compared to the other three methods, which can more accurately determine the magnitude of fault impact frequency and complete the separation of composite faults and unrelated frequencies. The detection of impact signal cycles is more accurate under high levels of background noise interference. When using this method to search for fault feature frequencies, it is possible to determine the fault feature frequency that is consistent with the actual working situation in a small iteration cycle. Not only does it avoid the error deconvolution problem caused by incorrect fault feature frequencies in the CYCBD method, but it also obtains more accurate periodic fault impact signals.

**Figure 6:**
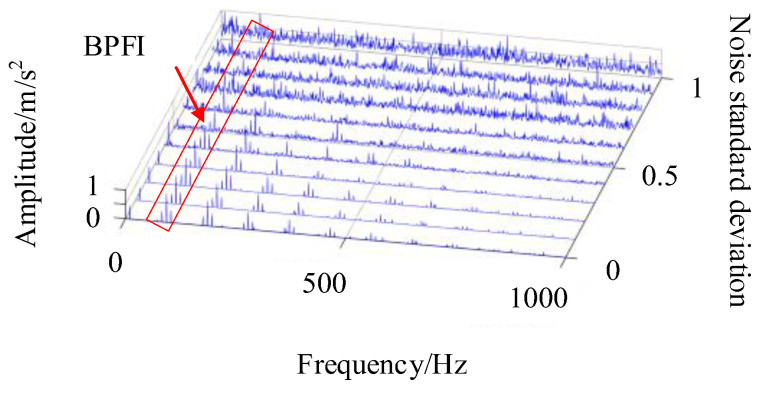
Improved envelope spectrum for inner ring faults.

In the simulation signal, the impact of bearing inner ring fault is weaker compared to the impact of the fault in the bearing’s outer ring, which increases the difficulty of detection. The detection of the inner circle fault cycle in the composite fault signal is shown in Figure 6. The fault feature frequency is 93 Hz. Since there are obvious sideband features in the envelope spectrum of the inner circle fault feature frequency, which are beneficial for locating the fault frequency resonance band, the range of features is selected to be 85~105 Hz. The obtained IES waterfall diagram is shown in Figure 6.

**Figure 7:**
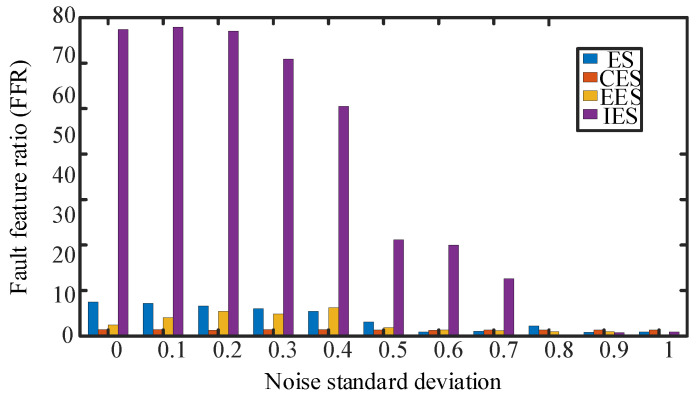
The effect of four periodic detection methods for inner ring faults.

In Figure 6, it is difficult to detect the inner circle fault feature frequency in the envelope spectrum with a noise standard deviation greater than 0.3, making it difficult to perform the correlation detection shown in Figure 4a. In the enhanced envelope spectrum shown in Figure 4c, the inner circle fault feature frequency is affected by other cyclostationary components in the signal, making it difficult to identify the fault frequency even under low signal-to-noise ratio conditions. In Figure 6, the enhanced envelope spectrum with a standard deviation of less than 0.5 can clearly show the fault frequency and its sideband characteristics in the inner ring bearing. But, as the noise signal increases, recognizing the sideband features becomes more difficult. Under the influence of noise signals with a standard deviation of 0.7, this method fails to identify the inner circle fault cycle, and the subsequent use of the CYCBD method is required to further suppress the noise level for feature extraction. When detecting the characteristic frequency of bearing inner ring faults, the FFR index size of each method is shown in Figure 7.

The IES has the ability to separate composite fault features that other methods do not possess, avoiding erroneous judgments when diagnosing fault impact periodicity, as shown in Figure 7. This method can achieve higher FFR indicators and improve the accuracy of fault cycle detection compared to other methods. It should also be noted that the detection performance of this method decreases when the impact signal is relatively weak compared to the noise signal. Therefore, it is necessary to use the CYCBD method to reduce noise interference and enhance the fault impact signal during the iteration process.

### 4.3. The IES-CYCBD Method Validation

**Figure 8:**
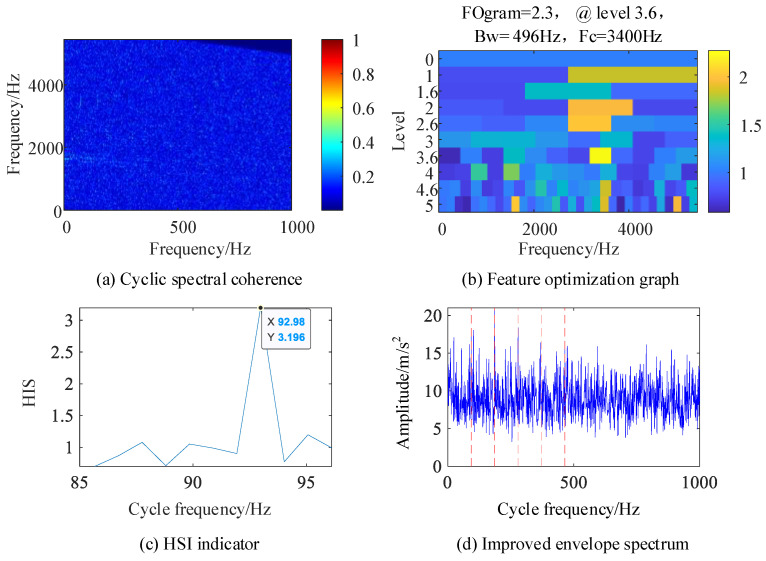
Composite fault simulation signal.

In order to verify the fault detection capability of the IES-CYCBD method under the influence of strong noise and strong harmonic signals, the inner ring fault feature frequency was separated from the bearing composite fault signal with a noise standard deviation of 1. The processing results are shown in Figure 8.

Figure 8a shows the cyclic spectral coherence image obtained using the fast spectral correlation algorithm for the original signal, and the resonance band is difficult to determine. Figure 8b is a feature optimization diagram that locates the frequency domain resonance band caused by bearing inner ring failure at the center frequency of 3400 Hz and the frequency band width of 496 Hz. In Figure 8d of the IES image of the original signal, the fault feature frequency is not clear, but it can be determined from Figure 8c that the cyclic feature frequency used for the CYCBD algorithm is 92.98 Hz, which is consistent with the inner circle fault feature frequency set by the simulation signal of 93 Hz, indicating that this method is more accurate in judging the cyclic feature frequency.

**Figure 9:**
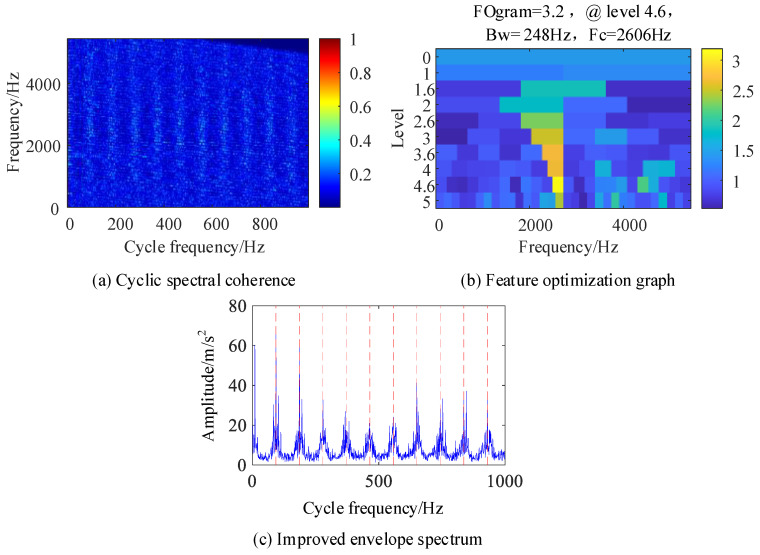
Signals after IES-CYCBD processing.

Figure 9 shows the results processed by the IES-CYCBD method. Figure 9a shows the cyclic spectral coherence map of the processed signal. Compared with Figure 8a, the light band formed by the characteristic frequency of bearing faults can clearly be seen. Figure 9b shows a feature optimization diagram of the processed signal. The figure shows that the optimal resonance frequency band corresponding to the inner circle fault is [2412, 2908] Hz, which is consistent with the resonance band excited by the inner circle set by simulation, indicating that the CYCBD method can improve the positioning of the resonance frequency band. Figure 9c shows the enhanced envelope spectrum separated from this frequency band, where the characteristic frequencies of inner circle faults are completely separated, demonstrating the ability of this method to accurately separate and diagnose fault types under high noise, composite faults, and other operating conditions.

## 5. Experimental Verification

In order to verify the accuracy of the proposed IES-CYCBD method for separating composite bearing faults, this paper designs a composite fault bearing test bench and verifies the algorithm using the vibration acceleration signal.

### 5.1. Bearing Composite Fault Test Signal

**Figure 10:**
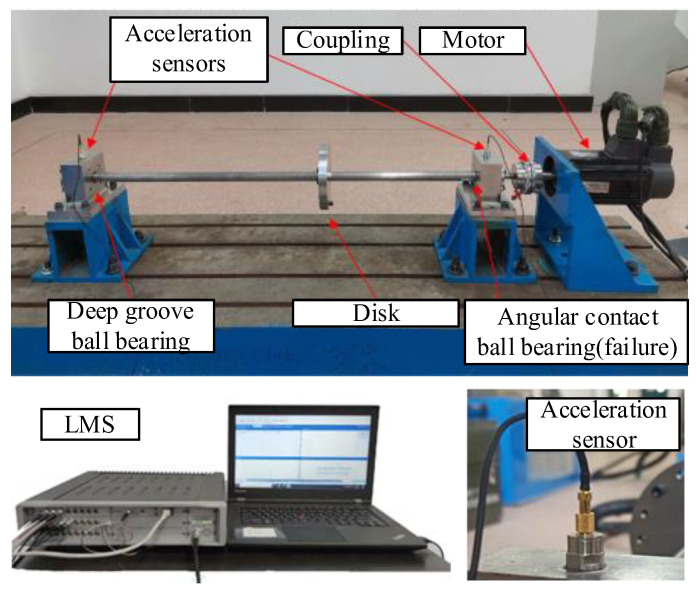
Rotor test bench.

**Figure 11:**
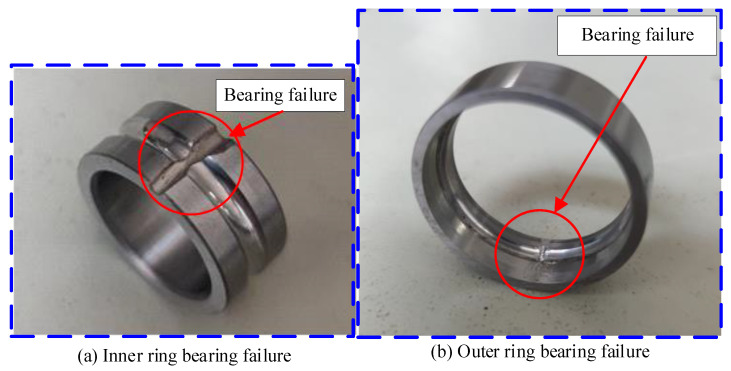
Faulty bearing.

The experimental device established in this paper is a bearing fault simulation rotor test bench, which is composed of a rotating shaft, a rotating disc, a cylindrical roller bearing (left fulcrum), an angular contact ball bearing (right fulcrum), a bearing support, an elastic coupling, a motor support, a motor, a base, etc. The structure of the rotor test bench is shown in Figure 10. The testing system used in the experiment includes a testing computer and acquisition software (Simcenter Testlab 2019.1) based on the LMS testing system. The overall testing system is shown in Figure 10. The sensitivity of the acceleration sensor used in the experiment is 100 mv/g. The faulty bearing model used in the experiment is a 7003AC angular contact ball bearing. The damage to the inner ring of the bearing is shown in Figure 11a, and the damage to the outer ring is shown in Figure 11b.

**Figure 12:**
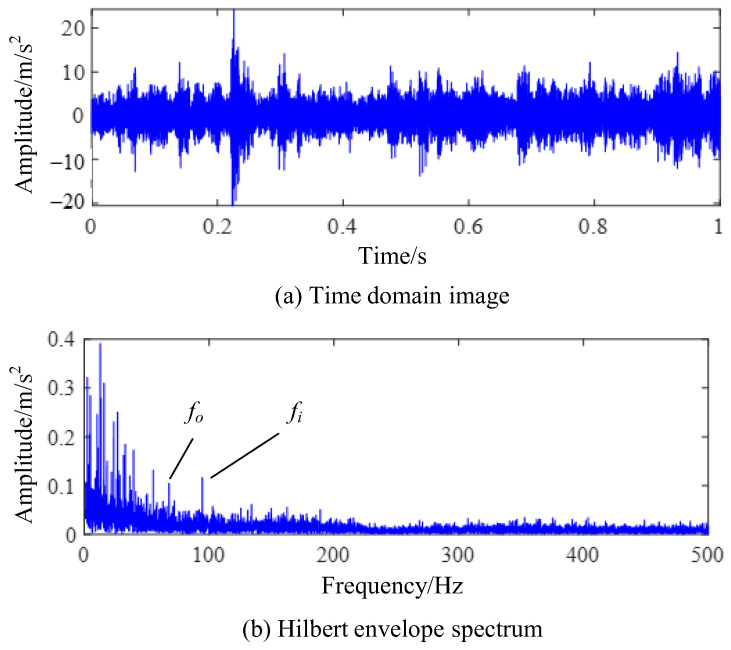
Test signal of compound fault experiment.

During the experiment, the residual value was set to1 × 10^−3^, the number of cycles was set to 500, and the rotation speed was set to 1000 r/min. With this rotation rate, the vibration signal is greatly affected by the low-frequency discrete frequency component. The time-domain image of bearing fault vibration signal collected through the testing system is shown in Figure 12a. In the figure, it can be seen that there are strong impact components in the time-domain signal. The bearing fault frequency calculated through the bearing structure is 117.4 Hz for the inner ring fault characteristic frequency and 82 Hz for the outer ring fault characteristic frequency. The envelope spectrum of the composite fault signal is shown in Figure 12b, and a frequency component similar to the two types of fault frequencies can be found in the figure. However, the harmonic component corresponding to this frequency component cannot be found in the envelope spectrum, making it difficult to accurately determine whether this frequency component is a fault characteristic frequency or a fault-independent frequency component.

### 5.2. Processing Results Obtained by IES-CYCBD Method

(1) Feature separation of inner ring faults

**Figure 13:**
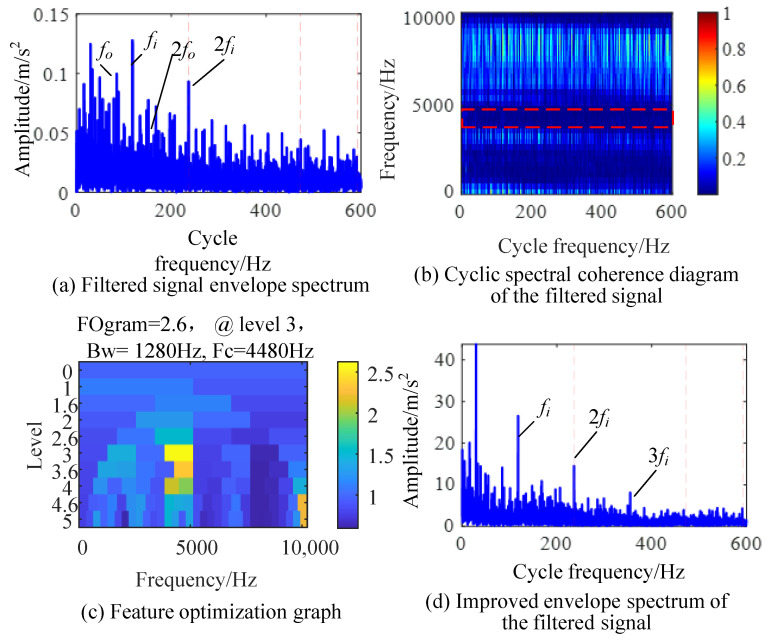
IES-CYCBD method for separating inner ring fault.

We used the IES to determine the accurate outer ring fault characteristic frequency of the test vibration signal by setting the search range of the fault characteristic frequency to [110, 120] Hz, and ultimately we obtained the actual inner ring fault characteristic frequency of *f_o_* = 118.3 Hz. The detection results are shown in Figure 13. 

We input the detected feature frequency group into the CYCBD method for filtering, and the envelope spectrum of the filtered signal is shown in Figure 13a. As the impact of inner circle faults in the signal dominates the vibration signal, the feature significance is relatively high and is only affected by discrete unrelated frequency components. A CSCoh image of the filtered signal is shown in Figure 13b; the highlighted part is mainly composed of discrete frequency components. According to the feature optimization in Figure 13c, the optimal demodulation frequency band is [3840, 5120] Hz. The IES after the demodulation of the frequency band is shown in Figure 13d; the frequency and harmonics of the inner ring fault characteristics can be clearly and accurately observed from the graph, indicating the successful separation of inner ring fault features from the signal.

(2) Feature separation of outer ring faults

**Figure 14:**
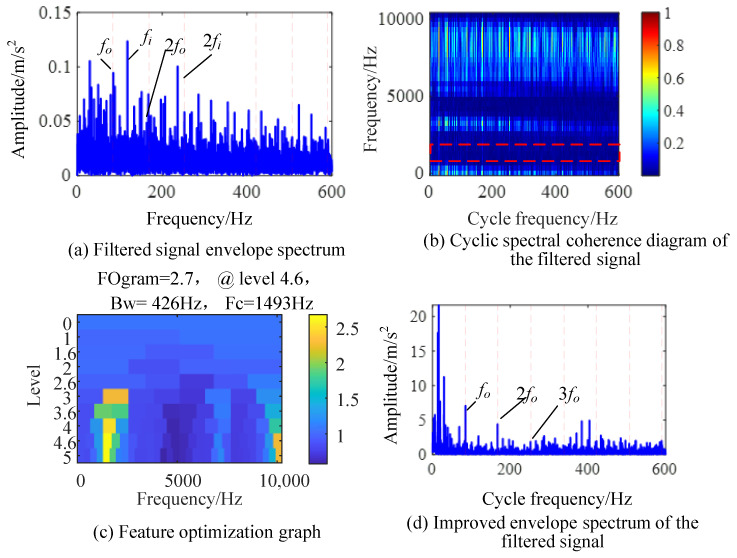
IES-CYCBD method for separating outter ring fault.

During the signal testing process, the impact of the outer ring fault is relatively small compared to the inner ring fault, so it is submerged in the fault-independent frequency and noise. The principle of the CYCBD method is to enhance the cyclostationary statistical features in the signal through filters. The cyclostationary characteristics of the inner ring fault impact will affect the separation of the outer ring fault features, as shown in Figure 14.

We set the search interval for faults to [70, 80] Hz, and used the harmonic product spectrum method to preliminarily determine that the actual characteristic frequency of bearing outer ring faults should be *f_i_* = 84.4 Hz. The envelope spectrum of the signal after deconvolution at this cyclic characteristic frequency is shown in Figure 14a. The signal envelope spectrum processed by the CYCBD method still has interference from the inner circle fault feature frequency, indicating that feature separation is ineffective, and the outer circle fault frequency is greatly affected by other frequency components, which affects accurate judgment. 

Figure 14b shows the cyclic spectrum coherent image of the signal, with the highlighted part mainly showing the dominant inner circle fault frequency in the filtered signal. At this time, the EES integrated in the full frequency band is not suitable for separating the outer circle fault frequency. A feature optimization diagram of the filtered signal is shown in Figure 14c, and the optimal demodulation resonance frequency band for outer circle faults is found to be [1280, 1706] Hz. The IES obtained by integrating the resonance frequency band is shown in Figure 14d, where the inner ring fault frequency is suppressed, and the fundamental and high-frequency harmonics of the outer ring fault characteristic frequency are more prominent. This successfully achieves the separation of outer ring fault features in composite faults.

### 5.3. Comparison of Resonance Frequency Band Positioning Methods

**Figure 15:**
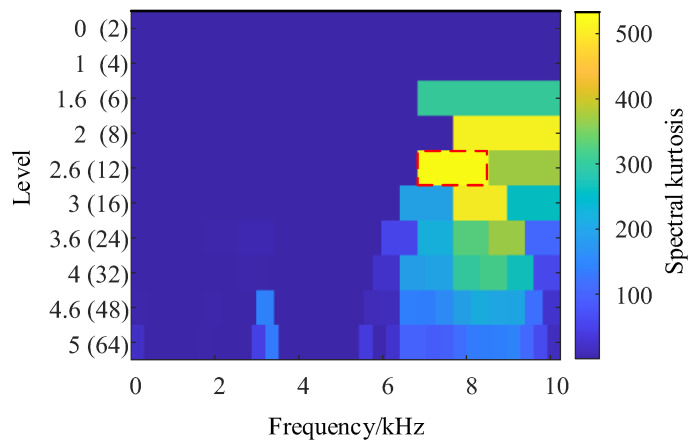
Fast spectral kurtosis.

**Figure 16:**
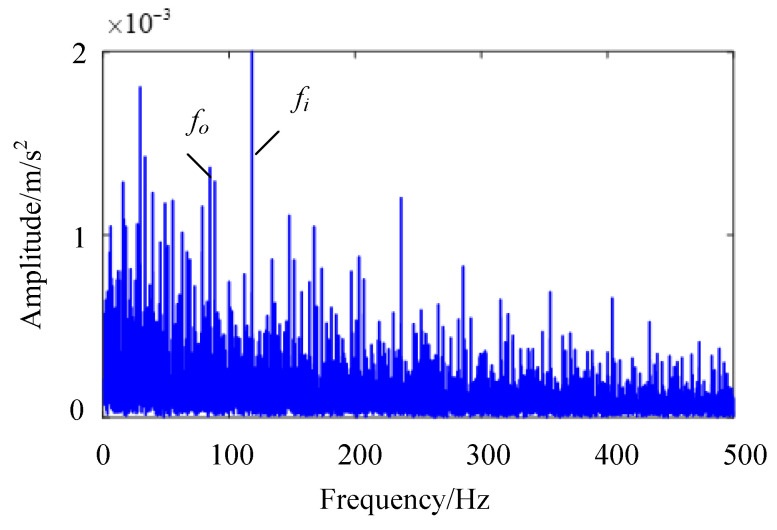
Envelope spectrum of bandpass filtered signal.

To verify the advantages of the IES-CYCBD method proposed in this chapter in separating composite fault features, a fast spectral kurtosis algorithm was used to analyze composite fault signals. The frequency band with the most prominent kurtosis value obtained through the fast spectral kurtosis method is [6826, 8533] Hz, as shown in Figure 15. The vibration signal is filtered using a bandpass filter, and the envelope spectrum of the bandpass filtered signal is shown in Figure 16.

In Figure 16, it can be observed that the fault characteristic frequency of the inner circle and the fault characteristic frequency of the outer circle still cannot be separated from the unrelated frequency components. The frequency of faults in both the inner and outer rings exists simultaneously and cannot be separated. This indicates that the fast spectral kurtosis method is not accurate enough to locate the resonance frequency band caused by bearing faults. By using bandpass filtering, bearing fault characteristic frequencies with dominant components can be identified, but it is difficult to separate other fault characteristic frequencies that are disturbed. By contrast, the IES-CYCBD method improves the signal-to-noise ratio of the signal due to its accurate frequency band positioning when separating bearing composite faults. Therefore, this method can effectively separate fault feature frequencies and accurately determine the location of defects in bearings.

## 6. Conclusions

Different types of bearing faults are coupled with each other, which increases the difficulty of fault diagnosis. Therefore, it is more important to propose a reasonable and effective composite fault feature separation method. In this paper, a feature decoupling method (IES-CYCBD) for bearing complex faults is proposed. The specific conclusions are as follows:

(1) An index composed of harmonic product spectrum kurtosis and entropy is proposed to locate the fault-excited resonance frequency band, and the frequency band is accurately located by using the feature optimization diagram. Compared with traditional envelope spectrum and enhanced envelope spectrum, the optimized IES has a better extraction effect on the target feature frequency.

(2) By using IES and the harmonic product spectrum, the problem of inaccurate cyclic feature vector in the filtering in CYCBD method is solved, the filtering effect of CYCBD is improved, and the periodic impact of target faults is strengthened.

(3) By separating different frequency bands from the filtered signal, the successful separation of complex fault features is realized. The proposed method is compared with the fast spectral kurtosis method, which shows that the IES-CYCBD method can accurately locate the bearing resonance band caused by faults and improve the significance of fault features.

However, further research is necessary due to the limitations of this method’s inability to achieve real-time processing and low algorithm adaptability in complex situations.

## Figures and Tables

**Table 1:** Simulation signal parameters of inner and outer ring fault.

Parameters	*N*_1_	*T_f_*	*f_f_*	*ξ_f_*	*A_f_*
Outer ring fault parameters	73	1/73	2600	1000	1
Inner ring fault parameters	93	1/93	3500	1000	0.5(1−cos(2π×10t))

## Data Availability

The raw data supporting the conclusions of this article will be made available by the authors on request.

## References

[1] Liew A., Feng N., Hahn E.J. (2002). Transient rotordynamic modeling of rolling element bearing systems. J. Eng. Gas Turbines Power.

[2] Qiu J., Seth B.B., Liang S.Y., Zhang C. (2002). Damage mechanics approach for bearing lifetime prognostics. Mech. Syst. Signal Process..

[3] Moazen Ahmadi A., Petersen D., Howard C. (2015). A nonlinear dynamic vibration model of defective bearings—The importance of modelling the finite size of rolling elements. Mech. Syst. Signal Process..

[4] Zmarzły P. (2020). Influence of bearing raceway surface topography on the level of generated vibration as an example of operational heredity. Indian J. Eng. Mater. Sci..

[5] Huang D., Liu Y., Liu H., Yi J. (2018). Resonances of elastic rotor induced by roller bearing with consideration of cage vibration. Proc. Inst. Mech. Eng. Part K J. Multi-Body Dyn..

[6] Borghesani P., Antoni J. (2018). A faster algorithm for the calculation of the fast spectral correlation. Mech. Syst. Signal Process..

[7] Buzzoni M., Antoni J., D’Elia G. (2018). Blind deconvolution based on cyclostationarity maximization and its application to fault identification. J. Sound Vib..

[8] Wang X., Yan X., He Y. (2020). Weak fault detection for wind turbine bearing based on ACYCBD and IESB. J. Mech. Sci. Technol..

[9] Chen B., Zhang W., Song D., Cheng Y. (2020). Blind deconvolution assisted with periodicity detection techniques and its application to bearing fault feature enhancement. Measurement.

[10] Randall R.B., Antoni J. (2011). Rolling element bearing diagnostics—A tutorial. Mech. Syst. Signal Process..

[11] Antoni J. (2007). Fast computation of the kurtogram for the detection of transient faults. Mech. Syst. Signal Process..

[12] Moshrefzadeh A., Fasana A. (2018). The Autogram: An effective approach for selecting the optimal demodulation band in rolling element bearings diagnosis. Mech. Syst. Signal Process..

[13] Peter W.T., Wang D. The sparsogram: A new and effective method for extracting bearing fault features. Proceedings of the 2011 Prognostics and System Health Managment Conference.

[14] Antoni J. (2016). The infogram: Entropic evidence of the signature of repetitive transients. Mech. Syst. Signal Process..

[15] Smith W.A., Fan Z., Peng Z., Li H., Randall R.B. (2016). Optimised Spectral Kurtosis for bearing diagnostics under electromagnetic interference. Mech. Syst. Signal Process..

[16] Barszcz T., JabŁoński A. (2011). A novel method for the optimal band selection for vibration signal demodulation and comparison with the Kurtogram. Mech. Syst. Signal Process..

[17] Randall R.B., Antoni J., Chobsaard S. (2001). The relationship between spectral correlation and envelope analysis in the diagnostics of bearing faults and other cyclostationary machine signals. Mech. Syst. Signal Process..

[18] Antoni J., Xin G., Hamzaoui N. (2017). Fast computation of the spectral correlation. Mech. Syst. Signal Process..

[19] Antoni J. (2007). Cyclic spectral analysis in practice. Mech. Syst. Signal Process..

[20] Kilundu B., Chiementin X., Duez J., Mba D. (2011). Cyclostationarity of Acoustic Emissions (AE) for monitoring bearing defects. Mech. Syst. Signal Process..

[21] Mauricio A., Smith W.A., Randall R.B., Antoni J., Gryllias K. (2020). Improved Envelope Spectrum via Feature Optimisation-gram (IESFOgram): A novel tool for rolling element bearing diagnostics under non-stationary operating conditions. Mech. Syst. Signal Process..

[22] Mauricio A., Qi J., Smith W.A., Sarazin M., Randall R.B., Janssens K., Gryllias K. (2020). Bearing diagnostics under strong electromagnetic interference based on Integrated Spectral Coherence. Mech. Syst. Signal Process..

[23] Mauricio A., Gryllias K. (2021). Cyclostationary-based Multiband Envelope Spectra Extraction for bearing diagnostics: The Combined Improved Envelope Spectrum. Mech. Syst. Signal Process..

[24] Xiao C., Tang H., Ren Y., Xiang J., Kumar A. (2021). A fault frequency bands location method based on improved fast spectral correlation to extract fault features in axial piston pump bearings. Measurement.

[25] Cui L., Du J., Yang N., Xu Y., Song L. (2019). Compound Faults Feature Extraction for Rolling Bearings Based on Parallel Dual-Q-Factors and the Improved Maximum Correlated Kurtosis Deconvolution. Appl. Sci..

[26] Jiang Y., Zhu H., Li Z. (2016). A new compound faults detection method for rolling bearings based on empirical wavelet transform and chaotic oscillator. Chaos Solitons Fractals.

[27] Hao Y., Song L., Cui L., Wang H. (2019). A three-dimensional geometric features-based SCA algorithm for compound faults diagnosis. Measurement.

[28] Xu Y., Chen J., Ma C., Zhang K., Cao J. (2019). Negentropy Spectrum Decomposition and Its Application in Compound Fault Diagnosis of Rolling Bearing. Entropy.

[29] Lyu X., Hu Z., Zhou H., Wang Q. (2019). Application of improved MCKD method based on QGA in planetary gear compound fault diagnosis. Measurement.

[30] Tang G., Tian T. (2020). Compound Fault Diagnosis of Rolling Bearing Based on Singular Negentropy Difference Spectrum and Integrated Fast Spectral Correlation. Entropy.

[31] Meng J., Wang H., Zhao L., Yan R. (2021). Compound fault diagnosis of rolling bearing using PWK-sparse denoising and periodicity filtering. Measurement.

[32] Saidi L., Ben Ali J., Fnaiech F. (2015). Application of higher order spectral features and support vector machines for bearing faults classification. ISA Trans..

[33] Manjurul Islam M.M., Kim J. (2019). Reliable multiple combined fault diagnosis of bearings using heterogeneous feature models and multiclass support vector Machines. Reliab. Eng. Syst. Saf..

[34] Dhiman H.S., Deb D., Carroll J., Muresan V., Unguresan M.L. (2020). Wind Turbine Gearbox Condition Monitoring Based on Class of Support Vector Regression Models and Residual Analysis. Sensors.

[35] Zhou H., Chen J., Dong G., Wang H., Yuan H. (2016). Bearing fault recognition method based on neighbourhood component analysis and coupled hidden Markov model. Mech. Syst. Signal Process..

[36] Jiang H., Li C., Li H. (2013). An improved EEMD with multiwavelet packet for rotating machinery multi-fault diagnosis. Mech. Syst. Signal Process..

[37] Zhang M., Jiang Z., Feng K. (2017). Research on variational mode decomposition in rolling bearings fault diagnosis of the multistage centrifugal pump. Mech. Syst. Signal Process..

[38] Chen J., Li Z., Pan J., Chen G., Zi Y., Yuan J., Chen B., He Z. (2016). Wavelet transform based on inner product in fault diagnosis of rotating machinery: A review. Mech. Syst. Signal Process..

[39] Wang Z., Zhou J., Du W., Lei Y., Wang J. (2022). Bearing fault diagnosis method based on adaptive maximum cyclostationarity blind deconvolution. Mech. Syst. Signal Process..

[40] Cheng Y., Chen B., Zhang W. (2019). Adaptive Multipoint Optimal Minimum Entropy Deconvolution Adjusted and Application to Fault Diagnosis of Rolling Element Bearings. IEEE Sens. J..

